# Bifurcations in a Model of Criminal Organizations and a Corrupt Judiciary

**DOI:** 10.3390/e26110906

**Published:** 2024-10-25

**Authors:** G. S. Harari, L. H. A. Monteiro

**Affiliations:** 1 Escola de Engenharia, Universidade Presbiteriana Mackenzie, São Paulo 01302-907, SP, Brazil; 2 Escola Politécnica, Universidade de São Paulo, São Paulo 05508-010, SP, Brazil

**Keywords:** backward bifurcation, corruption, dynamical system, justice, organized crime, population dynamics, transcritical bifurcation

## Abstract

Let a population be composed of members of a criminal organization and judges of the judicial system, in which the judges can be co-opted by this organization. In this article, a model written as a set of four nonlinear differential equations is proposed to investigate this population dynamics. The impact of the rate constants related to judges’ co-optation and ex-convicts’ recidivism on the population composition is explicitly examined. This analysis reveals that the proposed model can experience backward and transcritical bifurcations. Also, if all ex-convicts relapse, organized crime cannot be eradicated even in the absence of corrupt judges. The results analytically derived here are illustrated by numerical simulations and discussed from a crime-control perspective.

## 1. Introduction

Organized criminal groups are hierarchical structures composed of at least three persons who systematically plan and execute illegal activities to obtain financial benefits [1,2,3]. Mafia-type organizations exist in virtually every nation; hence, they are a worldwide problem. In 2023, more than 80% of the world’s population lived in countries with high criminality [3]. The countries with populations exceeding 100 million people that ranked in the top 25 of the Global Organized Crime Index 2023 included Brazil, China, India, Indonesia, Mexico, Nigeria, the Philippines, and Russia [3].

Typical illegal activities of organized crime include bank robbery, counterfeiting, money laundering, shipment hijacking, and trafficking of arms, drugs, and humans [3,4]. Criminal groups usually exert control over specific geographic regions [5]. There is relative impunity in these regions, which are used, for instance, to establish trafficking routes and distribution networks. Violence is often employed in their criminal actions and it also emerges in territorial disputes among rival groups. Hence, organized crime implies violence, which may escalate to homicides [4,5,6,7,8,9].

Corruption refers to the misuse of entrusted power to achieve illicit private gain [10,11]. Bribery, embezzlement, extortion, and nepotism are usual forms of corruption [12]. In 2016, the International Monetary Fund estimated the annual cost of bribery alone at about USD 2 trillion [13]. In 2019, a similar amount was lost in the world through corruption in health systems [14]. A common corrupt action involves making illegal payments to public agents in order to affect their decisions [15]. Corruption is seemingly a non-violent lawbreaking; however, it indeed kills. Examples can be found, for instance, in the healthcare sector [14,16,17] and in civil engineering [18]. Organized crime usually engages in corrupt practices to gain influence over central decision-makers, such as law enforcement officials, militaries, and politicians. In fact, corruption is a pathway through which criminal organizations perpetuate and extend their illegal endeavors [1,2].

The perceived level of public-sector corruption is regularly assessed by the non-governmental organization Transparency International. This organization computes the Corruption Perceptions Index (CPI), which varies from 0 to 100; that is, from highly corrupt to very clean [19]. For the countries mentioned above, the 2023 Global Organized Crime Index scores were as follows: Brazil—36, China—42, India—39, Indonesia—34, Mexico—31, Nigeria—32, the Philippines—34, and Russia—26 [19]. Notice that, for all these countries, the CPI is below the average.

Perhaps judicial corruption is the most detrimental form of corruption since it erodes public trust in the accessibility and impartiality of courts [10,11,20,21,22,23,24]. A corrupt judiciary compromises the fairness and integrity of the legal due process [10,11,20,21,22,23,24]. Unfortunately, judicial corruption linked to organized crime is a reality in several countries [25,26,27,28,29]. This illicit alliance violates human rights, promotes the impunity of lawbreakers, promotes the proliferation of criminal organizations, weakens adherence to the rule of law, and depletes the quality of governance [11,19].

Criminal behavior has been studied through mathematical approaches. For instance, there are mathematical models for the territorial expansion of gang activity [30], the emergence of spatiotemporal clusters of crime hotspots [31,32,33], the relationship between crime rates and incarceration rates [34,35], the effect of unemployment on crime incidence [36,37], the impact of technology on preventing crimes [38], the interaction between major and minor crimes [39,40], the individual’s decision to commit a crime based on the expected profit [41,42], the crime dynamics from a game-theoretic perspective [43], the influence of corruption in public procurement on economic growth [44], the payment of bribes to government bureaucrats [15], the effect of legal guns on the rate of crimes committed by illegal guns [45].

In addition, multi-compartment models have been proposed to describe the spread of criminal activity as a kind of social contagion [46,47,48,49,50,51,52]. In these models, the dissemination of criminal behavior is supposed to be analogous to the propagation of a contagious disease, because misconduct and infections can both be transmitted through social interactions [53]. A police force is taken into consideration in some studies [54,55]. The spread of corruption has also been examined by using epidemic-type models [56,57,58,59,60,61,62,63,64,65]. In these models, each individual in the population is usually classified as non-criminal (susceptible), criminal/corrupt, honest (immune), jailed, and recovered (from crime/corruption).

Here, corruption in the justice system is taken into account in a mathematical model of the spread of organized crime. This model is written a set of four nonlinear ordinary differential equations. This topic has been previously studied [66]; however, here, the growth of the criminal population is represented by the logistic equation, in order to impose a limitation on gang expansion. Also, in the model proposed here, a backward bifurcation can occur.

The remainder of this article is structured as follows. In Section 2, the model on gang formation and corruptible judiciary is introduced. In Section 3, the long-term dynamics of this model are analytically investigated by considering four distinct scenarios. In Section 4, the analytical results are depicted by computer simulations. Also, a real-world case of a criminal group is examined. In Section 5, the potential relevance of this study is discussed from a crime-control standpoint.

## 2. The Proposed Model

Let G(t), J(t), C(t), and P(t) be the densities (numbers of persons per area unit) of criminal gang members, non-corrupt judges, corrupt judges, and prisoners, respectively. Consider that the interaction among these individuals is described by the following nonlinear dynamical system:

(1) $$\begin{array}{ccc} \frac{dG}{dt} & = & \alpha G\left(\sigma -G\right)-\delta GJ+\beta P=\psi_{1}\left(G,J,P\right) \end{array}$$

(2) $$\begin{array}{ccc} \frac{dJ}{dt} & = & \gamma -\eta J-\rho GJ=\psi_{2}\left(G,J\right) \end{array}$$

(3) $$\begin{array}{ccc} \frac{dC}{dt} & = & \rho GJ-\varphi CJ=\psi_{3}\left(G,J,C\right) \end{array}$$

(4) $$\begin{array}{ccc} \frac{dP}{dt} & = & \delta GJ+\varphi CJ-\kappa P=\psi_{4}\left(G,J,C,P\right) \end{array}$$

In this model, αG(σ−G) is the well-known logistic equation, in which ασ is the growth rate constant and σ is the maximum density of gang members that can be sustainably maintained. The value of α depends on the level of intraspecific competition occurring within the gang, in which its members seek both prominence and the earnings derived from illegal activities. The value of σ is influenced by numerous factors, such as competition among rival groups, demand on the black market (for counterfeit products, drugs, weapons, etc.), the financial structure of the criminal group, geographical issues, judicial behavior, and police efforts in combating crime. The parameter σ is called carrying capacity in models of population dynamics [67].

Also, in these differential equations, δ is the rate constant of incarceration of gang members by non-corrupt judges, β is the rate constant at which former prisoners return to organized crime, γ is a constant influx of judges into the judiciary system, η is the rate constant of retirement/death of judges, ρ is the rate constant expressing the corruption of judges due to the contact with gang members, φ is the rate constant of incarceration of corrupt judges by non-corrupt judges, and κ is the rate constant of prisoner release/death, with β≤κ (because the rate at which gang members return to the criminal organization is either less than or equal to the rate at which they are released from jail). The nine parameters α, β, γ, δ, η, κ, ρ, σ, and φ are positive numbers.

Notice that the relationships between gang members and judges are described by the usual predator–prey terms found in the classic Lotka–Volterra model [67]. In terms of the parameter δ, the gang members are the prey and the judges are the predators; in terms of the parameter ρ, the roles are reversed. Notice also criminals are not sentenced to jail by corrupt judges, and there is no intraspecific competition among judges (since they act individually), as was considered to occur in gangs. In fact, in the judicial system, there is a steady inflow γ of judges and there are clear rules for career progression and retirement. In the model, the average length of a judge’s career is 1/η.

In the next section, the long-term behavior of this model is analytically investigated by using concepts taken from dynamic systems theory [68].

## 3. Analytical Results

A stationary solution of Equations (1)–(4) is written as $G\left(t\right)=G^{*}$, $J\left(t\right)=J^{*}$, $C\left(t\right)=C^{*}$, and $P\left(t\right)=P^{*}$, in which $G^{*}$, $J^{*}$, $C^{*}$, and $P^{*}$ are constants. These constants are obtained from dG/dt=0, dJ/dt=0, dC/dt=0, and dP/dt=0; that is, from $\psi_{1}\left(G^{*},J^{*},P^{*}\right)=0$, $\psi_{2}\left(G^{*},J^{*}\right)=0$, $\psi_{3}\left(G^{*},J^{*},C^{*}\right)=0$, and $\psi_{4}\left(G^{*},J^{*},C^{*},P^{*}\right)=0$. This stationary solution corresponds to an equilibrium point (a steady state) *E* with coordinates $\left(G^{*},J^{*},C^{*},P^{*}\right)$ in the state space G×J×C×P.

The local stability of the equilibrium point *E* can be inferred from the eigenvalues λ of the Jacobian matrix **J** computed at *E* [68]. This matrix is formed by the coefficients of the (linear) system obtained by linearizing the original nonlinear system around this point. If all eigenvalues of **J** have negative real parts, then *E* is locally asymptotically stable. If there is at least one eigenvalue with a positive real part, then *E* is unstable. If there is an eigenvalue with a null real part, then *E* can undergo a bifurcation [68]. Recall that the eigenvalues of **J** are obtained from det(J−λI)=0, where **I** represents the identity matrix.

For Equations (1)–(4), the Jacobian matrix computed at the equilibrium point *E* with coordinates $\left(G^{*},J^{*},C^{*},P^{*}\right)$ is as follows:

(5) $$J\left(G^{*},J^{*},C^{*},P^{*}\right)\;\;=\;\left[\begin{array}{cccc} a & b & 0 & \beta \\ c & d & 0 & 0\\ e & f & g & 0\\ h & j & \ell & -\kappa \end{array}\right]$$

in which $a=\alpha \sigma -2\alpha G^{*}-\delta J^{*}$, $b=-\delta G^{*}$, $c=-\rho J^{*}$, $d=-\eta -\rho G^{*}$, $e=\rho J^{*}$, $f=\rho G^{*}-\varphi C^{*}$, $g=-\varphi J^{*}$, $h=\delta J^{*}$, $j=\delta G^{*}+\varphi C^{*}$, and $\ell =\varphi J^{*}$. Since $G^{*}$, $J^{*}$, $C^{*}$, and $P^{*}$ represent constant densities, only non-negative values for these constants are taken into consideration in the analysis.

Here, four scenarios are examined, in which the values of ρ and β vary.

### 3.1. Case 1: ρ=0 and β=0

This case corresponds to the absence of judges’ co-optation by organized crime (that is, ρ=0) and the absence of recidivist ex-convicts (that is, β=0). In this ideal case, there are two equilibrium points called $E_{0}$ and $E_{1}$. Their coordinates are as follows:

(6) $$\begin{array}{ccc} E_{0} & = & \left(G_{0}^{*},J_{0}^{*},C_{0}^{*},P_{0}^{*}\right)=\left(0,\frac{\gamma }{\eta },0,0\right) \end{array}$$

(7) $$\begin{array}{ccc} E_{1} & = & \left(G_{1}^{*},J_{1}^{*},C_{1}^{*},P_{1}^{*}\right)=\left(\frac{(R_{0}-1)\gamma \delta }{\alpha \eta },\frac{\gamma }{\eta },0,\frac{\left(R_{0}-1\right){\left(\gamma \delta \right)}^{2}}{\alpha \kappa \eta^{2}}\right) \end{array}$$

in which we have the following:

(8) $$R_{0}=\frac{\alpha \eta \sigma }{\gamma \delta }$$

Notice that $E_{0}$ is a crime-free solution and $E_{1}$ is a corruption-free judiciary solution.

The eigenvalues of $E_{0}$ are $\lambda_{1}=\left(R_{0}-1\right)\gamma \delta /\eta$, $\lambda_{2}=-\eta <0$, $\lambda_{3}=-\gamma \varphi /\eta <0$, and $\lambda_{4}=-\kappa <0$. The eigenvalues of $E_{1}$ are $\lambda_{1}=\left(1-R_{0}\right)\gamma \delta /\eta$, $\lambda_{2}=-\eta <0$, $\lambda_{3}=-\gamma \varphi /\eta <0$, and $\lambda_{4}=-\kappa <0$. Thus, for $R_{0}<1$, $E_{0}$ is asymptotically stable and $E_{1}$ is unstable; for $R_{0}>1$, $E_{0}$ is unstable and $E_{1}$ is asymptotically stable. Therefore, $E_{0}$ and $E_{1}$ experience a transcritical bifurcation [68] for $R_{0}=1$, because these two equilibrium points, which have opposite stabilities, exchange their stabilities as $R_{0}$ varies around 1.

In demographic, ecological, and epidemiological studies, the parameter $R_{0}$ is called the basic reproduction number (or ratio) [69,70,71]. In such studies, $R_{0}<1$ implies the extinction of a species within an ecosystem or the eradication of a contagious disease from a city; $R_{0}>1$ leads to the preservation of the species or the persistence of the contagious disease [69,70,71]. Here, in case 1, suppression of organized criminal activity requires $R_{0}<1$; if $R_{0}>1$, such an activity endemically remains.

Regarding $R_{0}$, a sensitivity analysis via partial derivatives [72,73] shows the following:

(9) $$\begin{array}{ccc} & & \frac{\partial R_{0}}{\partial \alpha }\frac{\alpha }{R_{0}}=\frac{\partial R_{0}}{\partial \eta }\frac{\eta }{R_{0}}=\frac{\partial R_{0}}{\partial \sigma }\frac{\sigma }{R_{0}}=1 \end{array}$$

(10) $$\begin{array}{ccc} & & \frac{\partial R_{0}}{\partial \gamma }\frac{\gamma }{R_{0}}=\frac{\partial R_{0}}{\partial \delta }\frac{\delta }{R_{0}}=-1 \end{array}$$

Since the magnitudes of these calculations are equal, the parameters α, β, γ, η, and σ equally influence $R_{0}$. Evidently, $R_{0}$ increases with α, η, and σ, and it decreases with γ and δ.

### 3.2. Case 2: ρ=0 and β>0

In this case, there is no judge’s co-optation (that is, ρ=0), but ex-convicts can return to the criminal group (that is, 0<β≤κ). The equilibrium points are $E_{0}$ and $E_{2}$. The coordinates of $E_{2}$, representing the persistence of crime organized, are as follows:

(11) $$E_{2}=\left(G_{2}^{*},J_{2}^{*},C_{2}^{*},P_{2}^{*}\right)=\left(\frac{(R_{0}-1+\left(\beta /\kappa \right))\gamma \delta }{\alpha \eta },\frac{\gamma }{\eta },0,\frac{\left(R_{0}-1+\left(\beta /\kappa \right)\right){\left(\gamma \delta \right)}^{2}}{\alpha \kappa \eta^{2}}\right)$$

Notice that, if β<κ, then $G_{2}^{*}>G_{1}^{*}$ and $P_{2}^{*}>P_{1}^{*}$. Notice also that for β=κ (that is, total criminal recidivism), then $G_{2}^{*}=\sigma$. Therefore, for β=κ, the gang population can reach its maximum sustainable density, despite the actions of a non-corrupt judiciary.

For $E_{0}$, the eigenvalues are $\lambda_{1}$, $\lambda_{2}$, $\lambda_{3}=-\eta <0$, and $\lambda_{4}=-\gamma \varphi /\eta <0$, in which $\lambda_{1}$ and $\lambda_{2}$ are the roots of $\lambda^{2}+u_{1}\lambda +u_{2}=0$, with $u_{1}=\kappa +\left(\gamma \delta /\eta \right)-\alpha \sigma$ and $u_{2}=-\alpha \sigma \kappa +\left(\kappa -\beta \right)\gamma \delta /\eta$. Recall that the real part of the roots of the polynomial $\lambda^{2}+u_{1}\lambda +u_{2}=0$ is negative if $u_{1}>0$ and $u_{2}>0$. For β<κ, these conditions imply that $E_{0}$ is asymptotically stable if $R_{0}<1-\left(\beta /\kappa \right)$ and unstable if $R_{0}>1-\left(\beta /\kappa \right)$. For β=κ, $E_{0}$ is unstable (because $u_{2}<0$).

For $E_{2}$, the eigenvalues are $\lambda_{1}$, $\lambda_{2}$, $\lambda_{3}=-\eta <0$, and $\lambda_{4}=-\gamma \varphi /\eta <0$, in which $\lambda_{1}$ and $\lambda_{2}$ are the roots of $\lambda^{2}+v_{1}\lambda +v_{2}=0$, with $v_{1}=\alpha G_{2}^{*}+\kappa +\beta \gamma \delta /\left(\eta \kappa \right)$ and $v_{2}=\alpha \kappa G_{2}^{*}$. Therefore, $E_{2}$ is asymptotically stable if $G_{2}^{*}>0$; that is, if $R_{0}>1-\left(\beta /\kappa \right)$.

Thus, the crime-free steady-state solution $E_{0}$ and the endemic crime steady-state solution $E_{2}$ experience a transcritical bifurcation for $R_{0}=1-\left(\beta /\kappa \right)$. Observe that the critical value of $R_{0}$ in case 2 is lower than the critical value of $R_{0}$ in case 1 if β<κ. In fact, if ρ=0 and β<κ, organized crime is eradicated only if $R_{0}<1-\left(\beta /\kappa \right)$. If β=κ, this eradication is not possible.

### 3.3. Case 3: ρ>0 and β=0

This scenario is characterized by the absence of repeat ex-convicts (that is, β=0), but there are corrupt judges (that is, ρ>0). In this scenario, the equilibrium points are $E_{0}$, $E_{3}$, and $E_{4}$. The coordinates of $E_{3}$ and $E_{4}$, related to the entrenched presence of criminal organizations, are as follows:

(12) $$\begin{array}{ccc} E_{3} & = & \left(G_{3}^{*},\frac{\gamma }{\eta +\rho G_{3}^{*}},\frac{\rho G_{3}^{*}}{\varphi },\frac{\left(\delta +\rho \right)\gamma G_{3}^{*}}{\kappa (\eta +\rho G_{3}^{*})}\right) \end{array}$$

(13) $$\begin{array}{ccc} E_{4} & = & \left(G_{4}^{*},\frac{\gamma }{\eta +\rho G_{4}^{*}},\frac{\rho G_{4}^{*}}{\varphi },\frac{\left(\delta +\rho \right)\gamma G_{4}^{*}}{\kappa (\eta +\rho G_{4}^{*})}\right) \end{array}$$

in which we have the following:

(14) $$\begin{array}{ccc} G_{3}^{*} & = & \frac{-B+\sqrt{B^{2}-4AD}}{2A} \end{array}$$

(15) $$\begin{array}{ccc} G_{4}^{*} & = & \frac{-B-\sqrt{B^{2}-4AD}}{2A} \end{array}$$

with A=αρ/(γδ)>0, B=α(η−ρσ)/(γδ), and $D=1-R_{0}$. To find real numbers from Equations (14) and (15), then $B^{2}-4AD\geq 0$. This condition implies $R_{0}>1$ (that is, D<0) or $r\leq R_{0}<1$ with the following:

(16) $$r=\frac{4\eta \rho \sigma }{{\left(\eta +\rho \sigma \right)}^{2}}$$

Since $4mn/[{\left(m+n\right)}^{2}]\leq 1$ is the same as ${\left(m-n\right)}^{2}\geq 0$, which is always true for any real numbers *m* and *n*, then r≤1 (by taking m=η and n=ρσ). Therefore, if B<0 and $R_{0}<1$, $G_{3}^{*}$ and $G_{4}^{*}$ are positive real numbers if $r\leq R_{0}<1$; if B>0 and $R_{0}<1$, $G_{3}^{*}$ and $G_{4}^{*}$ are negative real numbers or complex conjugates with negative real parts; if B>0 and $R_{0}>1$, $G_{3}^{*}$ is the only positive real root; and if B≤0 and $R_{0}>1$, $G_{3}^{*}$ is again the only positive real root. In short, if $R_{0}>1$, only $G_{3}^{*}$ is positive; if $R_{0}<1$, $G_{3}^{*}$ and $G_{4}^{*}$ are positive if $R_{0}\geq r$. If $R_{0}=1$ (that is, D=0), $G_{3}^{*}=0$ and $G_{4}^{*}=-B/A$, which is positive if B<0.

For $E_{0}$, the eigenvalues are $\lambda_{1}=\left(R_{0}-1\right)\gamma \delta /\eta$, $\lambda_{2}=-\eta <0$, $\lambda_{3}=-\gamma \varphi /\eta <0$, and $\lambda_{4}=-\kappa <0$, as in case 1.

For $E_{3}$, the eigenvalues are $\lambda_{1}$, $\lambda_{2}$, $\lambda_{3}=-\varphi \gamma /\left(\eta +\rho G_{3}^{*}\right)<0$, and $\lambda_{4}=-\kappa <0$, in which $\lambda_{1}$ and $\lambda_{2}$ are the roots of $\lambda^{2}+p_{1}\lambda +p_{2}=0$, with $p_{1}=\eta +\left(\alpha +\rho \right)G_{3}^{*}$ and $p_{2}=\gamma \delta G_{3}^{*}\sqrt{B^{2}-4AD}$. Therefore, if $G_{3}^{*}$ is a positive real number, then $E_{3}$ is asymptotically stable (because $p_{1}>0$ and $p_{2}>0$).

For $E_{4}$, the eigenvalues are $\lambda_{1}$, $\lambda_{2}$, $\lambda_{3}=-\varphi \gamma /\left(\eta +\rho G_{4}^{*}\right)<0$, and $\lambda_{4}=-\kappa <0$, in which $\lambda_{1}$ and $\lambda_{2}$ are the roots of $\lambda^{2}+q_{1}\lambda +q_{2}=0$, with $q_{1}=\eta +\left(\alpha +\rho \right)G_{4}^{*}$ and $q_{2}=-\gamma \delta G_{4}^{*}\sqrt{B^{2}-4AD}$. Therefore, if $G_{4}^{*}$ is a positive real number, then $E_{4}$ is unstable (because $q_{2}<0$).

Notice that, for $R_{0}=r$, a backward bifurcation occurs, because at this value of $R_{0}$, the equilibrium points $E_{3}$ and $E_{4}$ with opposite stabilities emerge due to a saddle-node bifurcation [68]; in addition, there is the coexistence of the crime-free equilibrium point $E_{0}$ and the endemic crime equilibrium point $E_{3}$, and both are asymptotically stable for $r\leq R_{0}<1$. This bifurcation has been found in several studies on epidemiological systems [74,75,76,77]. For $R_{0}>1$, $E_{3}$ is the only attracting steady-state solution.

Observe that in case 1 (with ρ=0 and β=0), organized crime is eradicated if $R_{0}<1$; in case 3 (with ρ>0 and β=0), organized crime is eradicated only if $R_{0}<r<1$, which is a more restrictive condition. Thus, in cases 2 and 3, organized crime can persist even if $R_{0}<1$.

### 3.4. Case 4: ρ>0 and β>0

In the presence of judicial corruption (that is, ρ>0) and criminal relapse (that is, 0<β≤κ), the equilibrium points are $E_{0}$, $E_{5}$ and $E_{6}$. The coordinates of the endemic crime steady-state solutions are as follows:

(17) $$\begin{array}{c} E_{5}=\left(G_{5}^{*},\frac{\gamma }{\eta +\rho G_{5}^{*}},\frac{\rho G_{5}^{*}}{\varphi },\frac{\left(\delta +\rho \right)\gamma G_{5}^{*}}{\kappa (\eta +\rho G_{5}^{*})}\right) \end{array}$$

(18) $$\begin{array}{c} E_{6}=\left(G_{6}^{*},\frac{\gamma }{\eta +\rho G_{6}^{*}},\frac{\rho G_{6}^{*}}{\varphi },\frac{\left(\delta +\rho \right)\gamma G_{6}^{*}}{\kappa (\eta +\rho G_{6}^{*})}\right) \end{array}$$

in which we have the following:

(19) $$\begin{array}{c} G_{5}^{*}=\frac{-B+\sqrt{B^{2}-4AH}}{2A} \end{array}$$

(20) $$\begin{array}{c} G_{6}^{*}=\frac{-B-\sqrt{B^{2}-4AH}}{2A} \end{array}$$

with $H=1-R_{0}-\left(\beta /\kappa \right)\left(1+\left(\rho /\delta \right)\right)$. Both $G_{5}^{*}$ and $G_{6}^{*}$ are real roots if $R_{0}>r_{2}$ (that is, H<0) with the following:

(21) $$r_{2}=1-\frac{\beta }{\kappa }\left(1+\frac{\rho }{\delta }\right)$$

or if $r_{1}\leq R_{0}<r_{2}$ (that is, $B^{2}-4AH\geq 0$) with the following:

(22) $$r_{1}=rr_{2}$$

In this case, if $R_{0}>r_{2}$, $E_{5}$ in the only endemic crime equilibrium point with positive coordinates; if $R_{0}<r_{2}$, $E_{5}$ and $E_{6}$ have positive real coordinates if $R_{0}\geq r_{1}$. If $R_{0}=r_{2}$ (that is, H=0), $G_{5}^{*}=0$ and $G_{6}^{*}=-B/A$, which is positive if B<0.

Exactly determining the stability conditions of the equilibrium points for this case is challenging. To make this task more analytically tractable, this stability analysis is performed by considering ρ≪1 and φ≪1. Thus, judicial corruption and the incarceration of corrupt judges are supposed to happen at very low rates, which seems to be a plausible assumption.

First, assume that β<κ. In this scenario, the eigenvalues of $E_{0}$ are the same as those of $E_{0}$ in case 2. Thus, the crime-free equilibrium point is asymptotically stable if $R_{0}<1-\left(\beta /\kappa \right)$ and unstable if $R_{0}>1-\left(\beta /\kappa \right)$.

The eigenvalues of $E_{5}$ and $E_{6}$ are $\lambda_{1}=-\varphi J_{i}^{*}<0$ (with i=5,6) and the roots of the polynomial $\lambda^{3}+y_{1}\lambda^{2}+y_{2}\lambda +y_{3}=0$ with the following:

(23) $$\begin{array}{ccc} y_{1} & = & \alpha G_{i}^{*}+\eta +\frac{\beta \delta J_{i}^{*}}{\kappa }+\kappa \end{array}$$

(24) $$\begin{array}{ccc} y_{2} & = & \kappa \left(\alpha G_{i}^{*}+\eta \right)+\frac{\beta \delta \eta J_{i}^{*}}{\kappa }+\alpha G_{i}^{*}\left(\eta +\rho G_{i}^{*}\right)+\left(\frac{\beta }{\kappa }-1\right)\delta \rho G_{i}^{*}J_{i}^{*} \end{array}$$

(25) $$\begin{array}{ccc} y_{3} & = & \kappa \alpha G_{i}^{*}\left(\eta +\rho G_{i}^{*}\right)+\left(\beta -\kappa \right)\delta \rho G_{i}^{*}J_{i}^{*} \end{array}$$

The coefficients $y_{2}$ and $y_{3}$ can be rewritten as follows:

(26) $$\begin{array}{ccc} y_{2} & = & \kappa \left(\alpha G_{i}^{*}+\eta \right)+\frac{\beta \delta \eta J_{i}^{*}}{\kappa }+\gamma \delta G_{i}^{*}\left(2AG_{i}^{*}+B\right) \end{array}$$

(27) $$\begin{array}{ccc} y_{3} & = & \gamma \delta \kappa G_{i}^{*}\left(2AG_{i}^{*}+B\right) \end{array}$$

According to the Routh–Hurwitz criterion [78], the real part of the roots of $\lambda^{3}+y_{1}\lambda^{2}+y_{2}\lambda +y_{3}=0$ is negative if $y_{1}>0$, $y_{2}>0$, $y_{3}>0$, and $\Delta =y_{1}y_{2}-y_{3}>0$. From Equations (19) and (20), $2AG_{5}^{*}+B=\sqrt{B^{2}-4AH}$ and $2AG_{6}^{*}+B=-\sqrt{B^{2}-4AH}$. Observe that $y_{3}<0$ for $E_{6}$; hence, this equilibrium point is unstable. For $E_{5}$, $y_{1}>0$, $y_{2}>0$, $y_{3}>0$, and we have the following:

(28) $$\Delta =\left(\alpha G_{5}^{*}+\eta +\frac{\beta \delta J_{5}^{*}}{\kappa }\right)y_{2}+\kappa \left(\kappa \left(\alpha G_{5}^{*}+\eta \right)+\frac{\beta \delta \eta J_{5}^{*}}{\kappa }\right)>0$$

Thus, $E_{5}$ is asymptotically stable. Therefore, a backward bifurcation involving $E_{5}$ and $E_{6}$ can occur for $R_{0}=r_{1}$. A similar result was derived in case 3. Observe that Δ cannot be null; as a consequence, $E_{5}$ cannot experience a Hopf bifurcation [68].

For β=κ, $E_{0}$ is unstable as in case 2. Also, $E_{5}$ is the only equilibrium point with positive coordinates (since β=κ implies H<0 in Equations (19) and (20)) and it is asymptotically stable, because Equations (23)–(25) are reduced to the following:

(29) $$\begin{array}{ccc} y_{1} & = & \alpha G_{5}^{*}+\eta +\delta J_{5}^{*}+\kappa \end{array}$$

(30) $$\begin{array}{ccc} y_{2} & = & \kappa \left(\alpha G_{5}^{*}+\eta \right)+\alpha G_{5}^{*}\left(\eta +\rho G_{i}^{*}\right)+\delta \eta J_{5}^{*} \end{array}$$

(31) $$\begin{array}{ccc} y_{3} & = & \kappa \alpha G_{5}^{*}\left(\eta +\rho G_{5}^{*}\right) \end{array}$$

In addition, we have the following:

(32) $$\Delta =\left(\alpha G_{5}^{*}+\eta +\delta J_{5}^{*}\right)y_{2}+\kappa \left(\kappa \left(\alpha G_{5}^{*}+\eta \right)+\delta J_{5}^{*}\right)$$

As $y_{1}>0$, $y_{2}>0$, $y_{3}>0$, and Δ>0, then $E_{5}$ is an attracting steady state. Therefore, as in case 2, organized crime cannot be eradicated if β=κ.

The analytical results derived in this section are illustrated by the computer simulations presented in the next section.

## 4. Numerical Simulations

The proposed model was numerically solved by using the 4th-order Runge–Kutta method [79] with an integration time step of 0.01. Due to the scarcity of empirical data about organized crime and judicial corruption, most simulations were run with fictitious parameter values. These simulations illustrate the results obtained for cases 1 and 4; that is, for the ideal scenario (with β=0 and ρ=0) and the most realistic scenario (with β>0 and ρ>0). In these simulations, α (which affects the growth rate of the criminal organization) is taken as the bifurcation parameter. Thus, distinct steady states can be achieved by varying the value of α.

**Figure 1:**
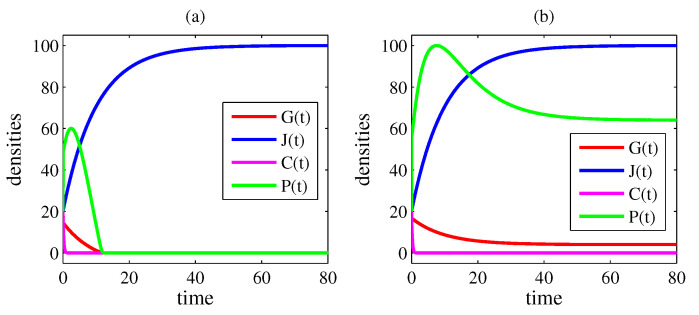
Time evolutions of G(t) (red line), J(t) (blue line), C(t) (magenta line), and P(t) (green line). The parameter values are γ=10, δ=8, η=0.1, κ=50, σ=20, and φ=0.2. In this case, β=0 and ρ=0. In (**a**), G(t)→0, J(t)→100, C(t)→0, and P(t)→0 for t→∞; in (**b**), G(t)→4, J(t)→100, C(t)→0, and P(t)→64 for t→∞. In (**a**), α=30 and $R_{0}=0.75<1$; in (**b**), α=50 and $R_{0}=1.25>1$. In this case, the criminal organization endures if $R_{0}>1$.

**Figure 2:**
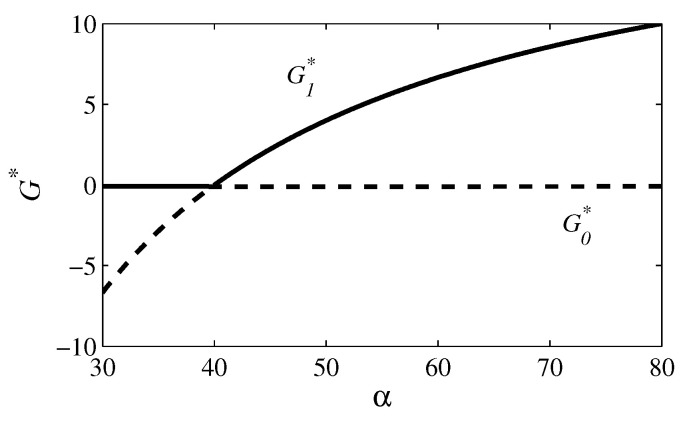
Bifurcation diagram for the parameter values employed in Figure 1. This diagram shows how $G_{0}^{*}$ and $G_{1}^{*}$ depend on α. The solid line represents an asymptotically stable solution and the dashed line represents an unstable solution. For α=40 (that is, $R_{0}=1$), a transcritical bifurcation occurs. Evidently, $G_{1}^{*}<0$ has no physical meaning.

**Figure 3:**
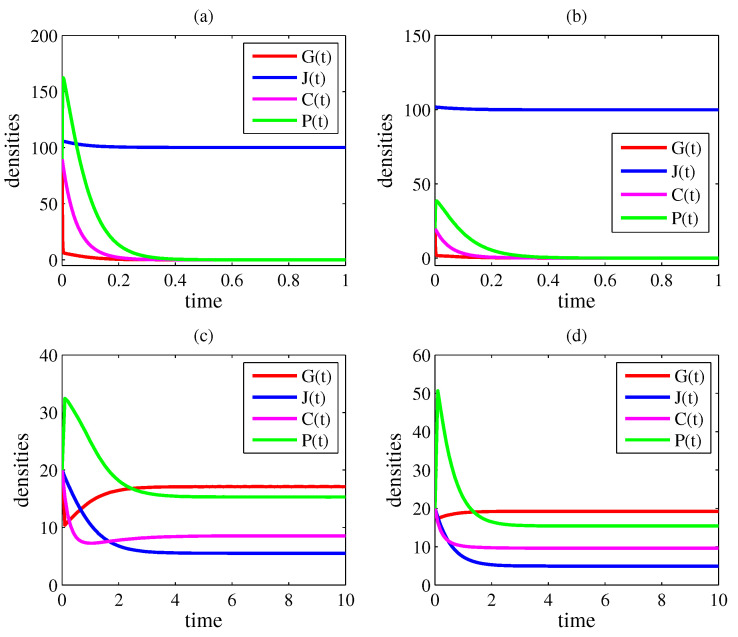
Time evolutions of G(t) (red line), J(t) (blue line), C(t) (magenta line), and P(t) (green line). The parameter values are γ=10, δ=8, η=0.1, κ=50, σ=20, and φ=0.2. In this case, β=30 and ρ=0.1. Therefore, $r_{1}=0.071$ and $r_{2}=0.393$. In (**a**,**b**), G(t)→0, J(t)→100, C(t)→0, and P(t)→0 for t→∞; in (**c**), G(t)→17.11, J(t)→5.52, C(t)→8.56, and P(t)→15.31 for t→∞; in (**d**), G(t)→19.22, J(t)→4.94, C(t)→9.61, and P(t)→15.40 for t→∞. In (**a**), α=2 and $R_{0}=0.050<r_{1}$; in (**b**,**c**), α=6 and $r_{1}<R_{0}=0.150<r_{2}$; in (**d**), α=20 and $R_{0}=0.500>r_{2}$. In this figure, organized crime persists either if $R_{0}>r_{2}$ or if $r_{1}<R_{0}<r_{2}$ and the initial condition pertains to the attraction basin of $E_{5}$. For $r_{1}<R_{0}<r_{2}$, the solution converges to $E_{0}$ if the initial condition pertains to the attraction basin of this equilibrium point. Thus, for $r_{1}<R_{0}<r_{2}$, the initial condition determines if the criminal organization will remain active.

**Figure 4:**
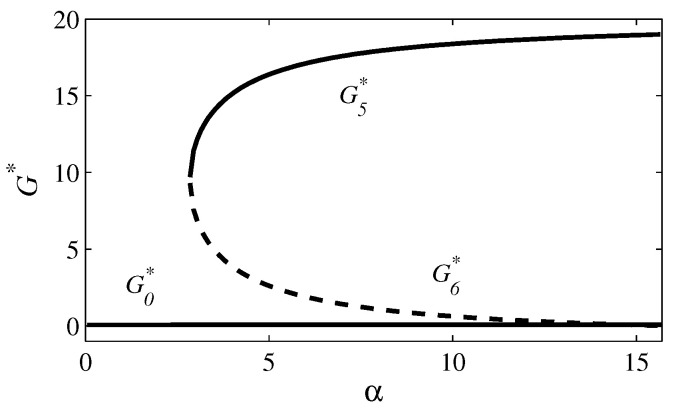
Bifurcation diagram for the parameter values used in Figure 3. This diagram shows how $G_{0}^{*}$, $G_{5}^{*}$, and $G_{6}^{*}$ depend on α. The solid line denotes an asymptotically stable solution and the dashed line denotes an unstable solution. For α=2.848 (that is, $R_{0}=r_{1}$), a backward bifurcation takes place.

In all figures, the parameter values are γ=10, δ=8, η=0.1, κ=50, σ=20, and φ=0.2. In Figure 1 and Figure 2, β=0 and ρ=0 (case 1); in Figure 3 and Figure 4, β=30 and ρ=0.1 (case 4). Figure 1 and Figure 3 exhibit the time evolutions of G(t) (red line), J(t) (blue line), C(t) (magenta line), and P(t) (green line). Figure 2 and Figure 4 present bifurcation diagrams of $G^{*}$ in the function of α, in which the solid line denotes an asymptotical stable steady state and the dashed line denotes an unstable steady state. The higher $G^{*}$, the greater the number of crimes and the lethal violence against society.

In Figure 1a, α=30 and $R_{0}=0.75<1$; in Figure 1b, α=50 and $R_{0}=1.25>1$. In Figure 1a, the variables converge to the coordinates of $E_{0}=\left(0,100,0,0\right)$ and, in Figure 1b, to the coordinates of $E_{1}=\left(4,100,0,64\right)$. In fact, for α=40 (that is, $R_{0}=1$), a transcritical bifurcation takes place, as shown in Figure 2. Thus, for α<40, the criminal organization is eradicated; for α>40, it continues to exist.

In Figure 3 and Figure 4, $r_{1}=0.071$ and $r_{2}=0.393$. In Figure 3a, α=2; therefore, $R_{0}=0.050$. Since $R_{0}<r_{1}$, there is a convergence to $E_{0}=\left(0,100,0,0\right)$; that is, the criminal group tends to disappear as time progresses (from any initial condition). In Figure 3b,c, α=6. Now, $R_{0}=0.150$ and $r_{1}<R_{0}<r_{2}$. In Figure 3b, there is a convergence to $E_{0}$ from the initial condition (G(0),J(0),C(0),P(0))=(20, 102, 20, 20) and in Figure 3c to $E_{5}=\left(17.11,\;5.52,\;8.56,\;15.31\right)$ from the initial condition (G(0),J(0),C(0),P(0))=(20, 20, 20, 20). Thus, for α=6, two attracting equilibrium points ($E_{0}$ and $E_{5}$) coexist, which is consistent with the occurrence of a backward bifurcation. In Figure 3d, α=20. In this plot, $R_{0}=0.500$. As $R_{0}>r_{2}$, there is a convergence to $E_{5}=\left(19.22,\;4.94,\;9.61,\;15.40\right)$; thus, organized crime persists (from any initial condition). Figure 4 exhibits the bifurcation diagram for β=30 and ρ=0.1. Notice that, for α=6, there are two attracting steady states, as observed in Figure 3b,c.

**Figure 5:**
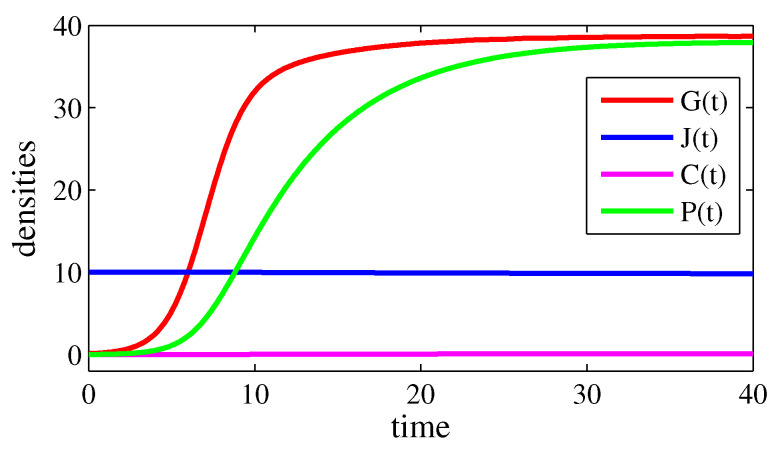
Time evolutions of G(t) (red line), J(t) (blue line), C(t) (magenta line), and P(t) (green line) in people/$\left(1000\;{\mathrm{km}}^{2}\right)$. The time *t* is given in years. The parameter values are α=1/40, β=1/6, γ=1/3, δ=1/50, η=1/30, κ=1/5, ρ= 1/40,000, σ=40, and φ=1/100. The initial condition is (G(0),J(0),C(0),P(0))=(10,0.1,0,0). In this case, G(t)→38.71, J(t)→9.72, C(t)→0.097, and P(t)→37.67, which are numbers consistent with a real-world scenario.

Now, consider the following real-world scenario (the references for the data presented in this paragraph were omitted in an effort to preserve our safety). First, assume that the time *t* is measured in years and that 1000 km^2^ corresponds to 1 area unit. Suppose that, in a region of a particular country, a judge dedicates 30 years to the career before retiring and there are approximately 10 judges per area unit. Thus, η=1/30 and $J^{*}≃10$; consequently, γ=1/3, by taking ρ = 1/40,000. Suppose also that about 1% of the judges are corrupt; that is, $C^{*}≃0.1$. In this region, there are about 40 members of a criminal organization per area unit; therefore, $G^{*}≃40$. If this density is near its possible maximum, then, σ=40 (if σ>40, the situation is even more serious). Since $\rho G^{*}=\varphi C^{*}$, then φ=1/100 (notice that ρ≪1 and φ≪1, as considered in case 4 of Section 3). The density of prisoners belonging to this criminal organization is assumed to be $P^{*}≃40$ (however, this number can be underestimated). When incarcerated, gang members spend an average of 5 years in jail; hence, κ=1/5. If 5/6 of the ex-convicts return to the criminal organization, then β=1/6. Also, since $P^{*}=\left(\delta +\rho \right)G^{*}J^{*}/\kappa$, then δ=1/50. Finally, assume that this organization started with G(0)=0.1 and, in 30 years, reached $G\left(30\right)≃G^{*}≃40$. This growth rate can be obtained by taking α=1/40. With these choices, $R_{0}=5$. Figure 5 exhibits the time evolution of the variables of the proposed model from the initial condition (G(0),J(0),C(0),P(0))=(10,0.1,0,0) (when the criminal organization was created at t=0, C(0)=0 and P(0)=0 because there were neither judges corrupted by it nor incarcerated gang members). In this plot, the variables converge to the coordinates of $E_{5}$. Despite the omission of references, this paragraph presents the rationale for obtaining parameter values from public databases [3,11,19,24].

## 5. Discussion and Conclusions

Corruption has a negative impact on democratic governance, economic growth, income distribution, and social development [12,80,81,82,83,84]. Judicial corruption perpetuates illicit activities and amplifies corruption throughout society; hence, it is the most harmful type of corruption [10,11,20,21,22,23,24]. Here, a mathematical model was proposed to investigate the interaction between a criminal organization and judges. In this model, corruption spreads via social contact. Four scenarios were analyzed in terms of ρ (the parameter related to judges’ co-optation) and β (the parameter related to criminal recidivism). Eradication of the criminal organization requires $R_{0}<1$ if ρ=0 and β=0; $R_{0}<1-\left(\beta /\kappa \right)$ if ρ=0 and β>0; $R_{0}<r$ if ρ>0 and β=0; and $R_{0}<r_{1}<r$ if ρ>0 and β>0. If ρ>0, a backward bifurcation can occur; therefore, eradication is also possible for $r<R_{0}<1$ if β=0 and for $r_{1}<R_{0}<r_{2}<1$ if β>0. However, in these cases of backward bifurcation, eradication depends on the initial condition (as shown in Figure 3b,c).

The lower the value of $R_{0}$, the lower the chances of the criminal organization persisting. Since $R_{0}=\alpha \eta \sigma /\left(\gamma \delta \right)$, then the value of this parameter can be reduced, for instance, by increasing δ (the incarceration rate of gang members) or by diminishing α (which is proportional to the growth rate of the organization) and σ (the maximum possible density of gang members). Nowadays, the advancing transnationalization of criminal organizations has increased α and σ, which has undermined the control of organized crime [1,2,3]. In fact, large-scale criminal networks have become even more professional and skilled in their operations [1,2,3]. Regrettably, systemic judicial corruption has strengthened the resilience of these criminal networks [10,19,22,24].

The higher ρ, the narrower the interval of $R_{0}$ within which the organized crime disappears. The value of ρ can be reduced by implementing accountability and transparency mechanisms, along with external controls on the judiciary [11,24].

The analyses also showed that if β=κ (all ex-convicts return to the criminal organization upon release), the criminal organization cannot be eradicated, even if ρ=0. The value of β is affected by the effectiveness of the programs dedicated to rehabilitating and reintegrating criminals. Therefore, it is crucial to diminish β; otherwise, organized crime will persist in society even if all judges are honest.

Perhaps the most controllable parameters for public authorities are the arrest rate for gang members (δ), the influx rate for judges entering the judicial system (γ), and the release rate for prisoners (κ). The mathematical expressions derived in this paper can be used to fit and forecast crime data, as well as to evaluate the impact of these three parameters, which can improve the management of public security policies. 

## Data Availability

The original contributions presented in the study are included in the article, further inquiries can be directed to the corresponding author.

## References

[1] Krishnan S. (2018). Organised crime—A threat to democracy. Int. J. Adv. Res..

[2] Reuter P., Paoli L. (2020). How similar are modern criminal syndicates to traditional mafias?. Crime Justice.

[3] Global Initiative Against Transnational Organized Crime (2023). Global Organized Crime Index 2023.

[4] Hauck P., Peterke S. (2010). Organized crime and gang violence in national and international law. Int. Rev. Red Cross.

[5] Lessing B. (2021). Conceptualizing criminal governance. Perspect. Polit..

[6] Oatley G., Crick T. Measuring UK crime gangs. Proceedings of the 2014 IEEE/ACM International Conference on Advances in Social Networks Analysis and Mining.

[7] Nsoesie E.O., Lima Neto A.S., Jay J., Wang H., Zinszer K., Saha S., Maharana A., Marinho F., Soares Filho A.M. (2020). Mapping disparities in homicide trends across Brazil: 2000–2014. Inj. Epidemiol..

[8] Dugato M., Calderoni F., Berlusconi G. (2020). Forecasting organized crime homicides: Risk terrain modeling of Camorra violence in Naples, Italy. J. Interpers. Violence.

[9] Cruz J.M., Vorobyeva Y. (2022). State presence, armed actors, and criminal violence in Central America. Sociol. Q..

[10] Rose-Ackerman S. (2001). Trust, honesty and corruption: Reflection on the state-building process. Arch. Eur. Sociol..

[11] Transparency International (2007). Global Corruption Report 2007.

[12] Hamdi H., Hakimi A. (2023). Corruption, imported innovation, and growth: Evidence using the panel smooth transition regression approach for developing countries. Reg. Sci. Policy Pract..

[13] International Monetary Fund (2016). Corruption: Costs and Mitigating Strategies. https://www.imf.org/en/Publications/Staff-Discussion-Notes/Issues/2016/12/31/Corruption-Costs-and-Mitigating-Strategies-43888.

[14] Garcia P.J. (2019). Corruption in global health: The open secret. Lancet.

[15] Rose-Ackerman S. (1975). The economics of corruption. J. Public Econ..

[16] Holmberg S., Rothstein B. (2011). Dying of corruption. Health Econ. Policy Law.

[17] Achim M.V., Väidean V.L., Borlea S.N. (2020). Corruption and health outcomes within an economic and cultural framework. Eur. J. Health Econ..

[18] Ambraseys N., Bilham R. (2011). Corruption kills. Nature.

[19] Transparency International (2024). Corruption Perceptions Index 2023. https://www.transparency.org/en/cpi/2023.

[20] Keyuan Z. (2000). Judicial reform versus judicial corruption: Recent developments in China. Crim. Law Forum.

[21] Hill J.N.C. (2010). Corruption in the courts: The Achilles’ heel of Nigeria’s regulatory framework?. Third World Q..

[22] Gloppen S., Søreide T., Williams A. (2014). Courts, corruption and judicial independence. Corruption, Grabbing and Development: Real World Challenges.

[23] Voigt S., Gutmann J. (2015). On the wrong side of the law - Causes and consequencesof a corrupt judiciary. Int. Rev. Law Econ..

[24] World Justice Project (2021). The World Justice Project Rule of Law Index 2021. https://worldjusticeproject.org/sites/default/files/documents/WJP-INDEX-2021.pdf.

[25] Zhilla F. (2011). Organised crime and judicial corruption in the Western Balkans. J. Financ. Crime.

[26] Brands H. (2011). Crime, irregular warfare, and institutional failure in Latin America: Guatemala as a case study. Stud. Confl. Terror..

[27] Prezelj I., Vogrincic N.O. (2020). Criminal and networked state capture in the Western Balkans: The case of the Zemun clan. South. Eur. Eur. Black Sea Stud..

[28] Rocha J.L., Rodgers D., Weegels J. (2023). Debunking the myth of Nicaraguan exceptionalism: Crime, drugs and the political economy of violence in a ‘narco-state’. J. Lat. Am. Stud..

[29] Raistenskis E., Krivins A., Aleksejeva L. (2023). Phehomenon of corruption in Albania: Towards cigarrete smugling. Access-Sci. Bus. Innov. Digit. Econ..

[30] Alsenafi A., Barbaro A.B.T. (2018). A convection-diffusion model for gang territoriality. Physica A.

[31] Short M.B., D’Orsogna M.R., Pasour V.B., Tita G.E., Brantingham P.J., Bertozzi A.L., Chayes L.B. (2008). A statistical model of criminal behavior. Math. Model. Meth. Appl. Sci..

[32] Zipkin J.R., Short M.B., Bertozzi A.L. (2014). Cops on the dots in a mathematical model of urban crime and police response. Discrete Contin. Dyn. Syst.-Ser. B.

[33] Calatayud J., Jornet M., Mateu J. (2023). Spatio-temporal stochastic differential equations for crime incidence modeling. Stoch. Environ. Res. Risk Assess..

[34] Gruszczynska B., Gruszczynski M. (2023). Crime and punishment-crime rates and prison population in Europe. Laws.

[35] Spelman W. (2008). Specifying the relationship between crime and prisons. J. Quant. Criminol..

[36] Mataru B., Abonyo O.J., Malonza D. (2023). Mathematical model for crimes in developing countries with some control strategies. J. Appl. Math..

[37] Soemarsono A.R., Fitria I., Nugraheni K., Hanifa N. (2021). Analysis of mathematical model on impact of unemployment growth to crime rates. J. Phys. Conf. Ser..

[38] Shukla J.B., Goyal A., Agrawal K., Kushwah H., Shukla A. (2013). Role of technology in combating social crimes: A modeling study. Eur. J. Appl. Math..

[39] Lacey A.A., Tsardakas M.N. (2016). A mathematical model of serious and minor criminal activity. Eur. J. Appl. Math..

[40] Park J., Kim P. (2021). Mathematical analysis of crime dynamics in and out of prisons. Math. Meth. Appl. Sci..

[41] Block M.K., Heineke J.M. (1975). A labor theoretic analysis of the criminal choice. Am. Econ. Rev..

[42] Caulkins J.P., Feichtinger G., Grass D., Hartl R.F., Kort P.M., Novak A.J., Seidl A., Wirl F. (2014). A dynamic analysis of Schelling’s binary corruption model: A competitive equilibrium approach. J. Optim. Theory Appl..

[43] Quinteros M.J., Villena M.J. (2022). On the dynamics and stability of the crime and punishment game. Complexity.

[44] Brianzoni S., Coppier R., Michetti E. (2011). Complex dynamics in a growth model with corruption in public procurement. Discrete Dyn. Nat. Soc..

[45] Monteiro L.H.A. (2020). More guns, less crime? A dynamical systems approach. Appl. Math. Comput..

[46] Sooknanan J., Bhatt B., Comissiong D.M.G. (2013). Catching a gang—A mathematical model of the spread of gangs in a population treated as an infectious disease. Int. J. Pure Appl. Math..

[47] McMillon D., Simon C.P., Morenoff J. (2014). Modeling the underlying dynamics of the spread of crime. PLoS ONE.

[48] Nyabadza F., Ogbogbo C.P., Mushanyu J. (2017). Modelling the role of correctional services on gangs: Insights through a mathematical model. R. Soc. Open Sci..

[49] Abbas S., Tripathi J.P., Neha A.A. (2017). Dynamical analysis of a model of social behavior: Criminal vs non-criminal population. Chaos Solitons Fractals.

[50] Srivastav A.K., Ghosh M., Chandra P. (2019). Modeling dynamics of the spread of crime in a society. Stoch. Anal. Appl..

[51] Srivastav A.K., Athithan S., Ghosh M. (2020). Modeling and analysis of crime prediction and prevention. Soc. Netw. Anal. Min..

[52] Opoku N.K.D., Bader G., Fiatsonu E. (2021). Controlling crime with its associated cost during festive periods using mathematical techniques. Chaos Solitons Fractals.

[53] Calderoni F., Comunale T., Campedelli G.M., Marchesi M., Manzi D., Frualdo N. (2022). Organized crime groups: A systematic review of individual-level risk factors related to recruitment. Campbell Syst. Rev..

[54] Nu no J.C., Herrero M.A., Primicerio M. (2008). A triangle model of criminality. Physica A.

[55] Misra A.K. (2014). Modeling the effect of police deterrence on the prevalence of crime in the society. Appl. Math. Comput..

[56] Abdulrahman S. (2014). Stability analysis of the transmission dynamics and control of corruption. Pac. J. Sci. Technol..

[57] Eguda F.Y., Oguntolu F., Ashezua T. (2017). Understanding the dynamics of corruption using mathematical modeling approach. Int. J. Innov. Sci. Eng. Technol..

[58] Kolokoltsov V.N., Malafeyev O.A. (2017). Mean-field-game model of corruption. Dyn. Games Appl..

[59] Shah N.H., Yeolekar B.M., Patel Z.A. (2017). Epidemics of corruption using incidence function. Econ. Comput. Econ. Cybern. Stud..

[60] Lemecha L., Feyissa S. (2018). Mathematical modeling and analysis of corruption dynamics. Ethiop. J. Sci. Sustain. Dev..

[61] Alemneh H.T. (2020). Mathematical modeling, analysis, and optimal control of corruption dynamics. J. Appl. Math..

[62] Danford O., Kimathi M., Mirau S. (2020). Mathematical modelling and analysis of corruption dynamics with control measures in Tanzania. J. Math. Inform..

[63] Fantaye A.K., Birhanu Z.K. (2022). Mathematical model and analysis of corruption dynamics with optimal control. J. Appl. Math..

[64] Jose S.A., Raja R., Alzabut J., Rajchakit G., Cao J., Balas V.E. (2022). Mathematical modeling on transmission and optimal control strategies of corruption dynamics. Nonlinear Dyn..

[65] Tesfaye A.W., Alemneh H.T. (2023). Analysis of a stochastic model of corruption transmission dynamics with temporary immunity. Heliyon.

[66] González-Parra G., Chen-Charpentier B., Kojouharov H.V. (2018). Mathematical modeling of crime as a social epidemic. J. Interdiscip. Math..

[67] Murray J.D. (2003). Mathematical Biology I: An Introduction.

[68] Guckenheimer J., Holmes P. (2002). Nonlinear Oscillations, Dynamical Systems, and Bifurcations of Vector Fields.

[69] Anderson R.M., May R.M. (1992). Infectious Diseases of Humans: Dynamics and Control.

[70] Heesterbeek J.A.P. (2002). A brief history of *R*_0_ and a recipe for its calculation. Acta Biotheor..

[71] Nishiura H., Inaba H. (2007). Discussion: Emergence of the concept of the basic reproduction number from mathematical demography. J. Theor. Biol..

[72] Saltelli A., Ratto M., Andres T., Campolongo F., Cariboni J., Gatelli D., Saisana M., Tarantola S. (2008). Global Sensitivity Analysis. The Primer.

[73] Ellner S.P., Guckenheimer J. (2011). Dynamic Models in Biology.

[74] Dushoff J., Huang W.Z., Castillo-Chavez C. (1998). Backwards bifurcations and catastrophe in simple models of fatal diseases. J. Math. Biol..

[75] van den Driessche P., Watmough J. (2000). A simple SIS epidemic model with a backward bifurcation. J. Math. Biol..

[76] Moraes A.L.S., Monteiro L.H.A. (2016). On considering the influence of recovered individuals in disease propagations. Commun. Nonlinear Sci. Numer. Simulat..

[77] Cui Q.Q., Qiu Z.P., Liu W.B., Hu Z.Y. (2017). Complex dynamics of an SIR epidemic model with nonlinear saturate incidence and recovery rate. Entropy.

[78] Ogata K. (2001). Modern Control Engineering.

[79] Griffiths D.F., Higham D.J. (2010). Numerical Methods for Ordinary Differential Equations: Initial Value Problems.

[80] Gupta S., Davoodi H., Alonso-Terme R. (2002). Does corruption affect income inequality and poverty?. Econ. Gov..

[81] Gründler K., Potrafke N. (2019). Corruption and economic growth: New empirical evidence. Eur. J. Polit. Econ..

[82] Lawless W.F. (2019). The interdependence of autonomous human-machine teams: The entropy of teams, but not individuals, advances science. Entropy.

[83] Mongi C., Saidi K. (2023). The impact of corruption, government effectiveness, FDI, and GFC on economic growth: New evidence from global panel of 48 middle-income countries. J. Knowl. Econ..

[84] Zang L.Z., Zhang B.Q., Xiong F. (2023). Multimodal assessment of political corruption worsening national poverty: Evidence of mediating and moderating effects from global panel data. Chin. Public Adm. Rev..

